# Influence of material composition on the morphology and engineering properties of waste plastic binder composite for construction purposes

**DOI:** 10.1016/j.heliyon.2022.e11207

**Published:** 2022-10-22

**Authors:** Yusuf Babatunde, John Mwero, Raphael Mutuku, Yinusa Jimoh, Daniel Oguntayo

**Affiliations:** a Pan African University, Institute for Basic Sciences, Technology and Innovation (PAUSTI), Juja, Kenya; b Department of Civil Engineering, University of Ilorin, Ilorin, Nigeria; c Landmark University SDG 11(Sustainable Cities and Communities Research Group), Nigeria; d Department of Civil Engineering, Landmark University, Omu-aran, Nigeria

**Keywords:** Waste PET plastic, PET-Quary dust composite, Structural lightweight concrete, Recycling, Strength

## Abstract

Ways of mitigating the menace caused by the abundance of waste plastic generated have been a global concern. Efforts are geared towards intensification of the recycling culture for circular economy. Recent studies combined waste plastic and sand to make Waste Plastic Binder (WPB) composite materials. However, sand mining operation has been associated with environmental and ecological issues. This study explores the engineering properties of waste plastic and quarry dust composite for sustainable infrastructural development. The polyethylene terephthalate type of plastic (PET) was employed, and which was melted and mixed with QD at different compositions of 1:0, 1:1, 1:2, and 1:3 respectively. The influence of the varying compositions on the morphological and engineering properties of resulting WPB composite was investigated. The scanning electron microscopy image showed that WPB composite with higher percentage of QD possess lesser pore space, and which influenced the high strength values. The findings showed P&QD 1:3 have highest compressive strength value of 20.1MPa, and which meets up with the American Concrete Institute and South African standard minimum requirement of 17MPa for structural lightweight concrete applicable for walkways, walling and water retaining structures constructions.

## 1 Introduction

The global increase in plastic production and demand from the growing population has led to the increasing trend of the number of generated plastics, annually (Umasabor& Daniel, 2020; Awolusi et al., 2022). Global plastic production rise from 1.5 million tonnes in 1950 to 365 million tonnes in 2019 (KARSLIOĞLU, 2021; Li et al., 2020); with about 8 million tonnes of these plastic wastes ending up in oceans and which has led to the death of millions of wildlife and affected 700 species (Getor et al., 2020). According to Dumbili and Henderson (2020), Nigeria contributes between 0.13–0.34 million tonnes of waste plastic into the marine environment. This has posed a major threat to the survival of environmental habitats or ecosystems. For instance, aquatic organisms swallowed small-sized plastic particles which get into the food chain from their system which is detrimental to both humans and animals. In addition, plastics are entangled in aquatic organisms such as seabirds, turtles, dolphins among others, and obstruct breathing, resulting in death (Kehinde et al., 2020; Kumar et al., 2021). However, the current management culture to mitigate the unprecedented rate of waste plastic is poor, most especially, in developing countries. Hence, research should be focused on ways to sustainably address the menace of waste plastic.

Several studies have been carried out to ascertain the suitability and potentiality of waste plastic as construction materials. In those studies, waste plastic was explored as a partial replacement of aggregate in a concrete mix. Low strength properties were reported to have characterized the produced concrete and the higher the contents of waste plastic in the concrete mix, the lower the strength properties (Almeshal et al., 2020; Belmokaddem et al., 2020; Boucedra et al., 2020). Therefore, fewer proportions of waste plastic can effectively be incorporated in a concrete mix without a significant reduction in concrete strength (Mustafa et al., 2019; Needhidasan et al., 2020). Furthermore, research has shown that waste plastic can serves as a binder in composite (Aina et al., 2022; Campanhão et al., 2022; da Silva et al., 2021; Tene et al., 2019; Babatunde et al., 2022). For instance, Aneke et al. (2021) study the morphological properties and durability of high-strength performance bricks made from a combination of PET waste (PW) and foundry sand (FS). PET waste bricks (PWBs) were made by varying the percentage of PET to FS (PW:FS) to 20%, 30%, and 40% of the dry mass of FS. The strength of plastic bricks made from a blend of 30 percent PW and 70 percent foundry sand was reported to be 36.18 MPa, which is about 60.31 percent higher than that of fired clay bricks. They also reportedly absorbed less water and retained their maximum strengths even after being completely submerged in water and acidic concentrations. They came to the conclusion that one of the sustainable ways of recycling the numerous plastic wastes, which results in the preservation of soil, water, and space, is the use of PET waste and foundry as raw materials for the production of masonry bricks.

The aforementioned research has shown that a sustainable way of managing PET wastes is its utilization in the construction industry. Therefore, this study explores the engineering properties of waste plastic and quarry dust composite for sustainable infrastructural development. Morphological, density, compressive strength, water absorption, ultrasonic pulse velocity, and dynamic modulus of elasticity properties of the composite at different proportions of melted PET plastic and quarry dust are evaluated, results analyzed, and compared with standard specifications for lightweight concrete.

## 2 Materials and methods

### 2.1 Waste plastic

**Figure 1:**
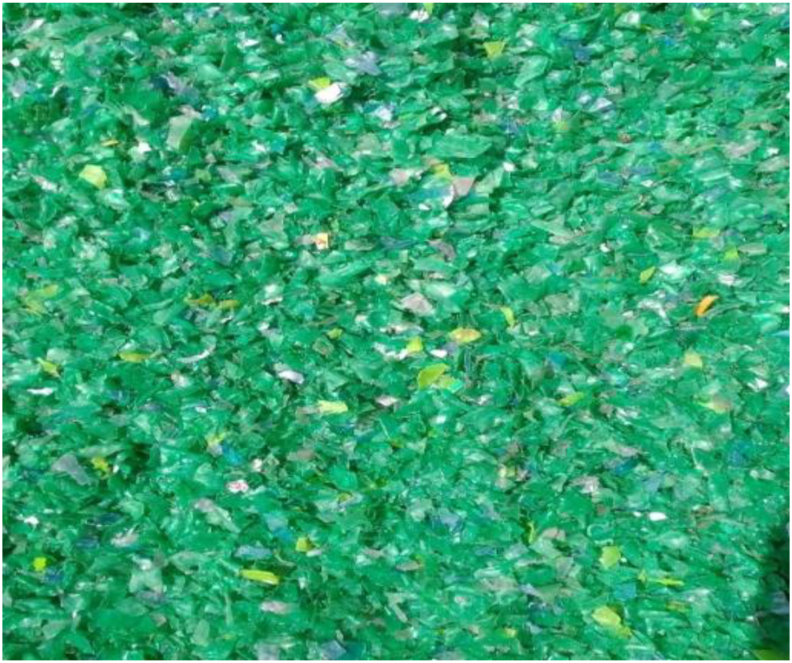
Pulverized waste PET Plastic.

The Polyethylene terephthalate (PET) type of waste plastic was employed for this study. PET plastic is the most commonly generated waste plastic and it dominates waste plastic streams. The waste plastic was procured from a plastic recycling outlet in an Industrial area, in Nairobi, Kenya. Collected waste plastic was sorted and pulverized into small sizes as shown in Figure 1. The waste plastic was thoroughly washed and dried. The physical properties of the waste plastic such as specific gravity, water absorption, and density were determined and presented in Table 1.

**Table 1:** Physical properties of plastic and quarry dust materials.

S/N	Property	Plastic	Quarry Dust
1.	Specific gravity	1.12	2.48
2.	Water absorption (%)	0	2.96
3.	Density (g/cm^3^)	1.02	1.32

### 2.2 Quarry dust

**Figure 2:**
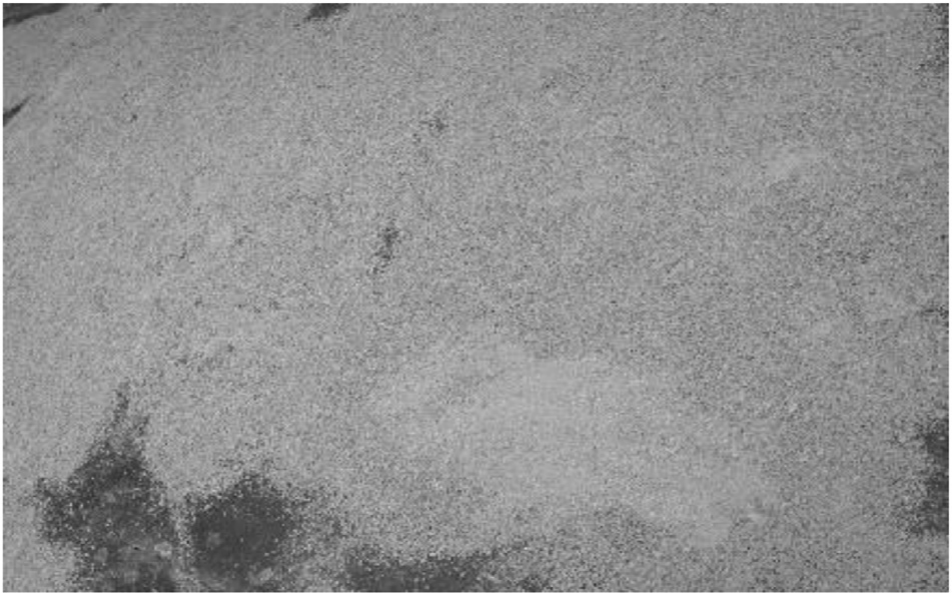
Quarry dust.

**Figure 3:**
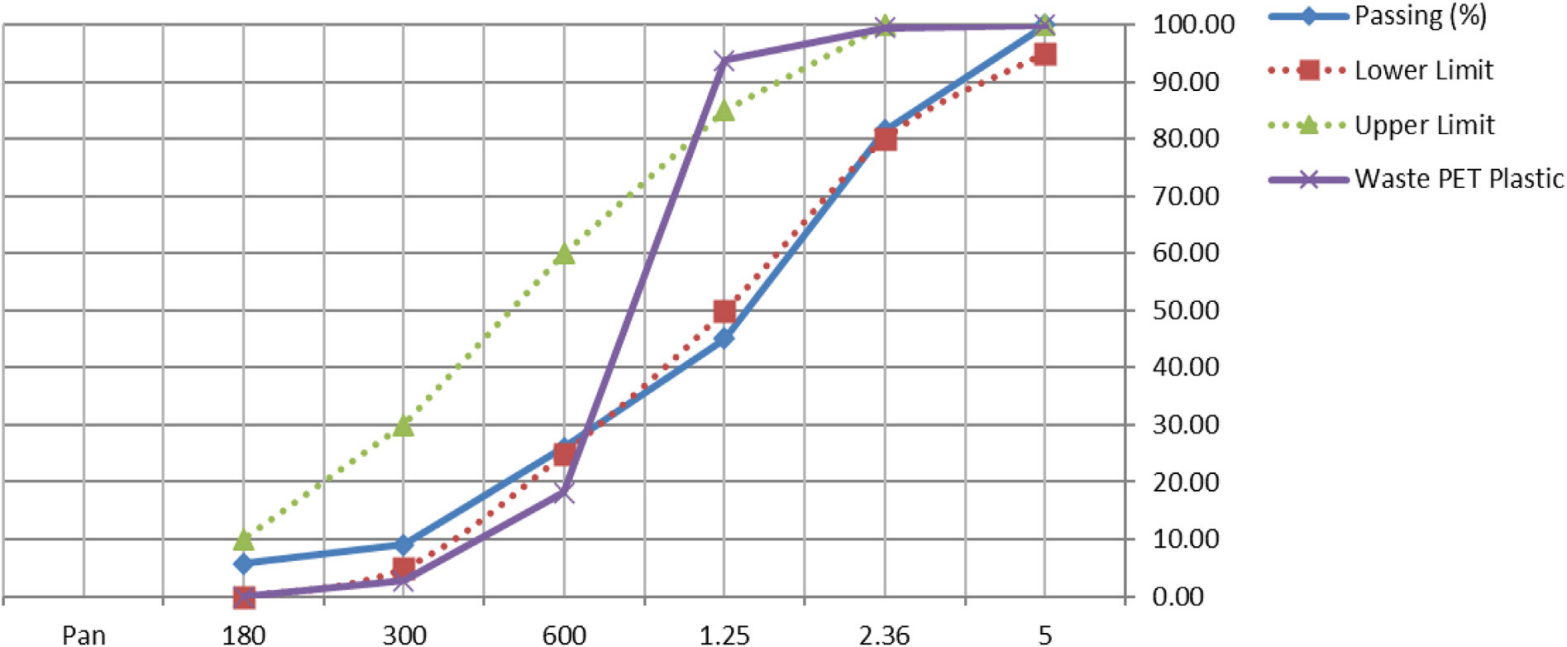
Particle size distribution of quarry dust materials.

The quarry dust or manufactured sand was locally sourced from aggregate and construction materials company at Kasarani, Kenya. The quarry dust was ensured to be free from any impurities. It was sieved through a 2.36 mm sieve and material that passes through the sieve was employed for the study. Afterward, sieve analysis, specific gravity, and density of the quarry dust were performed in compliance with ASTM C136; and ASTM C128–01 and presented in Figures 2 and 3, and Table 1.

### 2.3 Sample preparation and laboratory investigations

**Figure 4:**
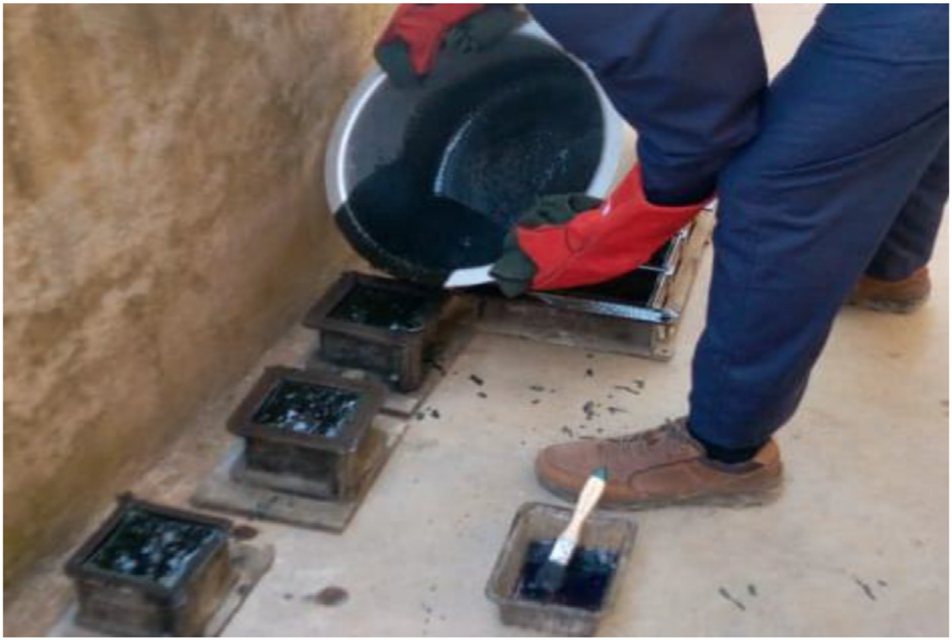
Filling of moulds with WPB composite.

**Figure 5:**
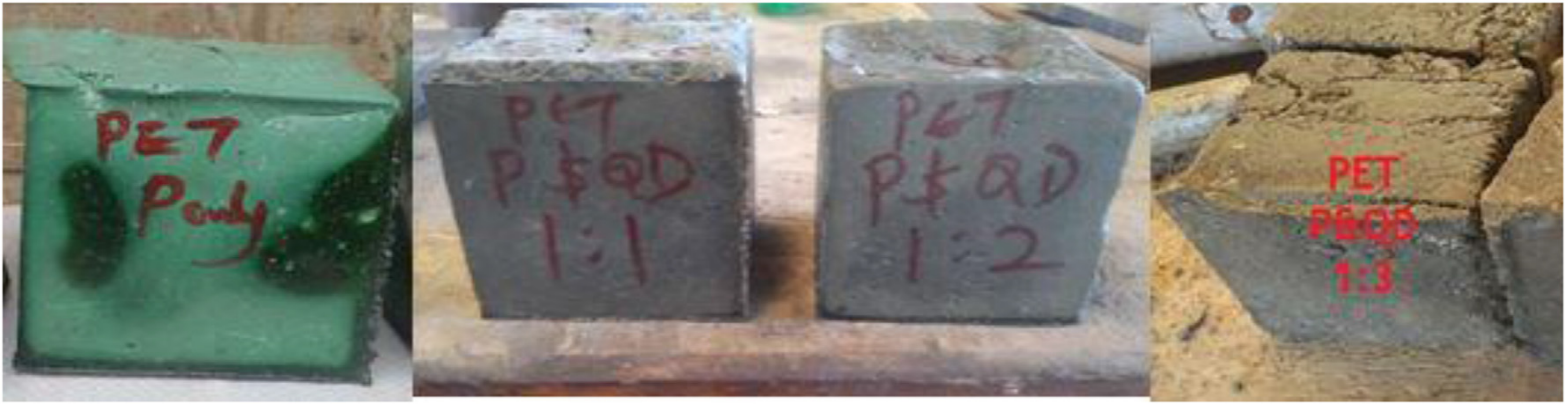
Samples of WPB composites (P&QD; 1:1, 1:2, & 1:3).

The PET plastic (P) was combined with Quarry dust (QD) at three different proportions of 1:0, 1:1, 1:2, and 1:3 respectively. The constituents’ materials were batch by weight. The appropriate weight of the waste plastic was batched into an aluminium container, and then transferred into an Electric furnace at 250^o^c. It was allowed to melt for 45 min. Afterward, it was removed, and already batched quarry dust was added to the viscous waste plastic gradually. The materials were stirred continuously to give a homogenous and uniform mortar-like composite. In the advent that the materials have not mixed well and it was sticky, the composite is returned to the Electric furnace for 10–20 min. The duration for the waste plastic to melt in the Electric furnace depends on the quantity of the material. After the materials have mixed well and become homogenous which gives a mortar-like composite, then, it was transferred into 100 × 100 × 100 mm steel moulds as shown in Figure 4. The composite was allowed to cool for 1 h at a laboratory temperature of 25^o^c. Then, it was demoulded and allowed to cool further for 48 h. As seen in Figure 5, the green colour is a result of the green pigment present in the waste PET plastic. Though, the composite was black at molten state, the colour transit to dark color for the composite after cooling, and it became darker as the quarry dust increases as shown in Figure 5.

However, the density of the hardened waste plastic binder composite was determined in accordance with ASTM C138/C138M–17a. An average of three density values measurements of different mix ratios represents the density for each mix. Samples were subjected to a uniaxial compression test in accordance with ASTM C39/C39M–18. The test was done at a loading rate of 1 mm/min to record the variable applied force and its corresponding deformation till failure or complete disintegration. The mechanical test was repeated three times for each mix to ensure that the results are repeatable. JEOL JCM–7000 benchtop Scanning Electron Microscopy (SEM) with Neoscope software was employed to assess the morphological properties of the composite. Waste plastic binder composite samples for each mixing ratios was cut into 20 mm thickness size. It was placed on an aluminium sample holder and transferred into the machine. The observation condition was set to low vacuum and acceleration voltage of 15kv. The images were taken at different speeds and magnifications for analysis. The non-destructive test was conducted on the waste plastic binder composite of different mix compositions using an Ultrasonic concrete tester, model C369N, CE 20 Matest, Italy. It was first calibrated to 0.45 μsecs. An indirect method of testing was employed and the travel time for the signal from the transducer to the receiver was recorded. The ultrasonic velocity was computed as the ratio of the travel distance to time. The test was conducted in accordance with ASTM C597–16. The dynamic modulus of elasticity was computed using Eq. (1) (Belmokaddem et al., 2020).

(1) $$E_{d}=\left(V^{2}.\rho /g\right)$$

Where E_d_ = Dynamic modulus of elasticity (GPa) ρ = actual density (Kg/m^3^).

V = UPV (km/s) g = acceleration due to gravity (9.81 m/s^2^).

## 3 Results and discussion

### 3.1 Influence of material composition on morphological properties

**Figure 6:**
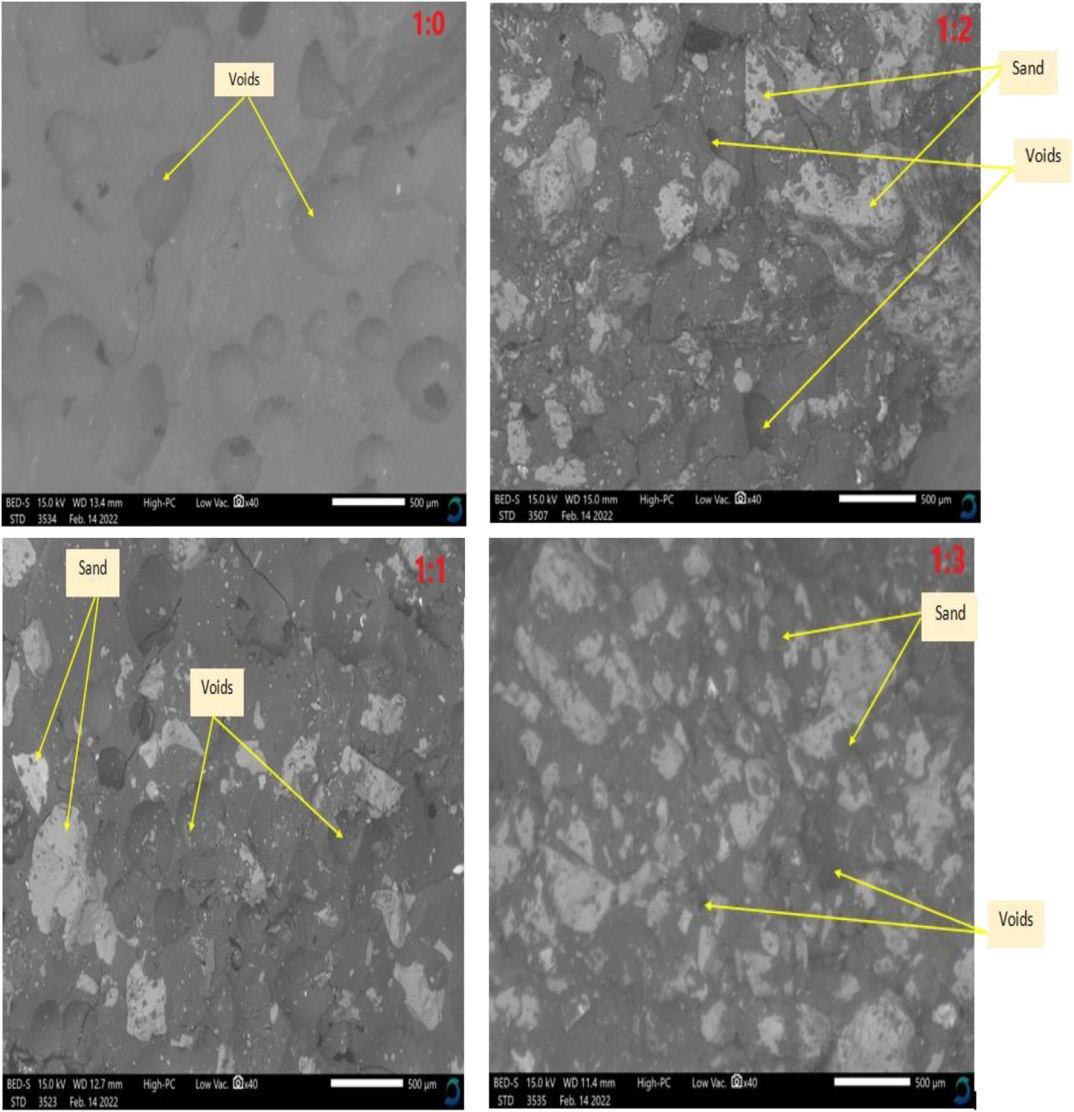
SEM Images of WPB composites.

**Figure 7:**
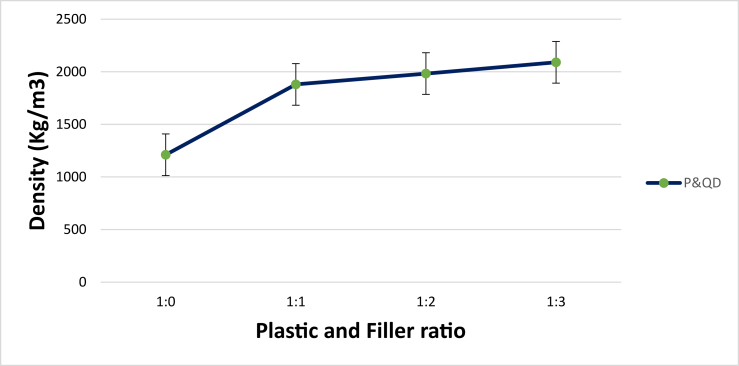
Influence of material composition on density of WPB composites.

Figure 6 showed the scanning electron microscopy images of the WPB composite at different mix compositions. P&QD 1:0 shows a large presence of voids as can be seen in Figure 7. This can be attributed to the escaped entrapped air. During production, the air ingress due to the steam and got trapped in the molten plastic, which escaped at the cooling phase, thereby leaving voids. The presence of pores can endanger and depreciate the unit weight and compressive strength of composites (Belmokaddem et al., 2020). The addition of quarry dust tends to fill the voids, and the higher the dosage of quarry dust, the lesser the voids. The voids diminish as the content of quarry dust increases. It can be observed that the quarry dust clustered in the plastic and the size distribution interlocks well and which could produce some reinforcement and consequently improve the strength of the composite. A similar observation was reported by Aneke et al. (2021), plastic waste bricks with a higher presence of foundry sand gave minimal pore space and consequently possessed greater strength. They identified chemical compositions of carbon C, hydrogen H, and Oxygen O, on the surface of the plastic waste bricks, and which was said to be rendered by PET plastic, while the foundry sand rendered the dominant elemental compound of Si, Fe, and Al observed. Not only that the quarry dust fill voids, it also prevents cracks widen and linkage.

### 3.2 Influence of material composition on density

The strength of a material, in mechanical terms, is greatly influenced by its density in the hardened state. High strength, low porosity, and a small number of voids are often attributed to denser cemented material (Thiam et al., 2021). The density of WPB composite at different mix ratios is presented in Figure 7. As observed, the density increases as the content of quarry dust increases. Quarry dust has a higher density than plastic. Compare to the control WPB composite at ratio 1:0, WPB composite of P&QD 1:1, P&QD 1:2, and P&QD 1:3 rise by 55, 64, and 73 % respectively. The recommended density of lightweight concrete (LWC) is within the threshold of 500–1900 kg/m^3^ (Agyeman et al., 2019). All the WPB composites mixes are within the specified threshold except P&QD 1:3 which is slightly above the recommended values.

### 3.3 Influence of material composition on compressive strength

**Figure 8:**
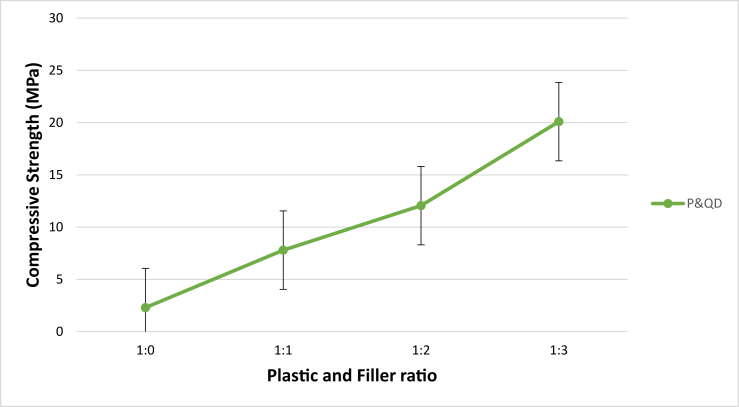
Influence of material composition on the Compressive Strength of WPB composites.

The compressive strength defines the resistance of WPB composite to failure under axial forces. It provides the property needed by Engineers to assess the behaviour of materials throughout service conditions. Figure 8 presents the compressive strength of WPB composite at different mix proportions. As shown, the strength of the composite increases as the content of quarry dust increases. The quarry dust reinforced the strength properties of the WPB composite. However, the final strength of the WPB composite depends on the intrinsic strength and percentage of the filler material. Compare to P&QD 1:0, the strength values increase by 70.5, 80.9, and 88.6% for P&QD 1:1, P&QD 1:2, and P&QD 1:3 respectively. A similar increase in compressive strength of Plastic Waste Binder (PWB) increases as the percentage level of filler materials increases was also observed by (Agyeman et al., 2019; Akinwumi et al., 2019; Aneke et al., 2021; Thiam et al., 2021; Babatunde et al., 2022). The morphology of the viscoelastic and ductile properties of plastic waste as its melts under temperature enabled it to form a stronger composite matrix, tension resistance, and developed high strength. The application of waste PET plastic in composite bricks production improves the compressive and tensile strength of the composite, and as well contributes to a 40 % reduction of CO_2_ emissions (Aneke et al., 2021). Agyeman et al. (2019) attributed the improvement to the adhesive strength between the filler particles and waste plastic surface area. However, Thiam et al. (2021) opined that the strength is due to the high degree of crystallinity of polymers as a result of the different polymer branches, which increases as the polymer solidifies or cools. It is noteworthy that, the compressive strength value of 20.1MPa for P&QD 1:3 meets up with the American Concrete Institute (ACI) and South African Standard (SANS 227:2007) minimum requirement of 17MPa for structural lightweight concrete. Thus, the WPB composite can find application as load-bearing walls and other retaining structures.

### 3.4 Influence of material composition on water absorption

**Figure 9:**
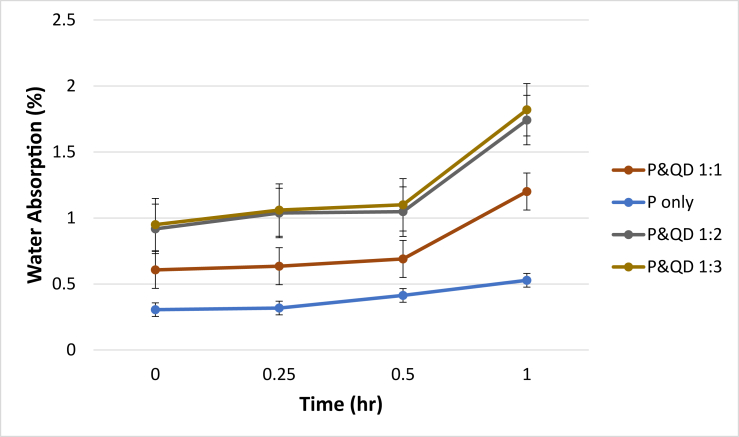
Influence of material composition on the Water Absorption of WPB composites.

Water absorption test was carried out to gain insight into the pore structure and durability of the plastic samples. It is an indirect method to assess the coarseness or fineness of the sample pore structure or porosity. The permeation ability of a porous material depends on the pore structure. In other words, the ease at which fluids flow into and through the porous medium (Thiam et al., 2021). As can be seen in Figure 9, the water absorption increases as the duration of the samples in water increases. It can be said that water ingress and occupied the voids present in the WPB composite P&QD 1:0 samples. Plastics are hydrophobic materials, which reveals the nearly zero water absorption property of WPB composite P&QD 1:0 samples, while quarry dust is a hydrophilic material, which influenced the higher water absorption of P&QD 1:1, 1:2, and 1:3. The level of absorption depends on the number of pores available on the surface of materials, which explains the increase of water absorption of WPB composite as the content of quarry dust increases. The water absorption obtained is in agreement with that of Jacob-Vaillancourt &Sorelli (2018), who attributed the low water absorption to the hydrophobic nature of waste PET plastics. Generally, the water absorption of all the WPB composite is far below 10%, which is the maximum recommended for concrete.

### 3.5 Influence of material composition on ultrasonic pulse velocity

**Figure 10:**
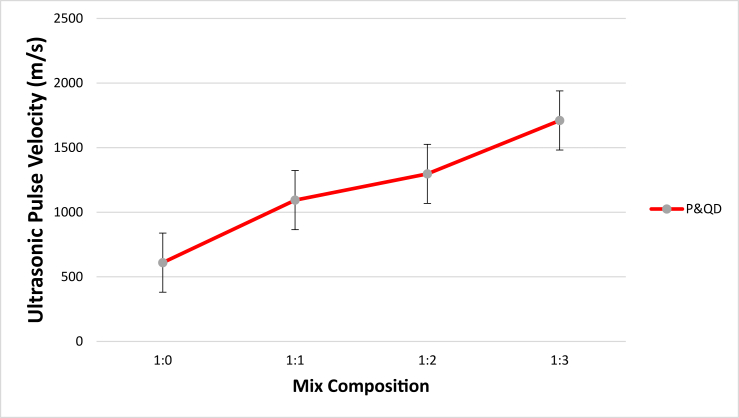
Influence of material composition on the Ultrasonic Pulse Velocity of WPB composites.

**Figure 11:**
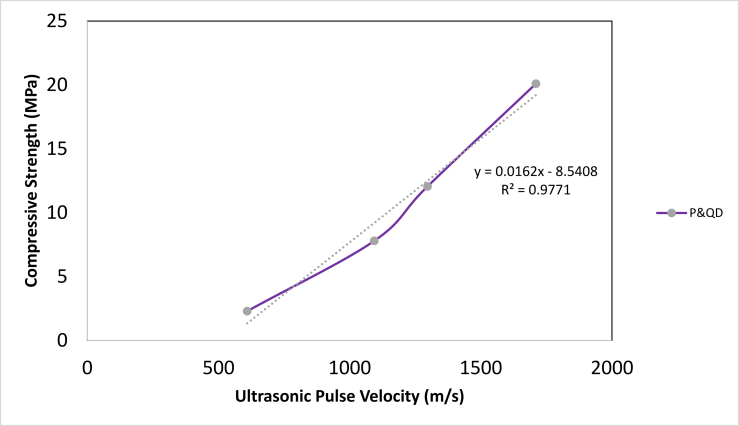
Relationship between ultrasonic pulse velocity and compressive strength of WPB composite.

The Ultrasonic Pulse Velocity (UPV) reveals information on the rate of transfer of pulse in WPB composite specimens. Indirectly, it provides inside into the porosity of specimens. Figure 10 shows the results of the UPV of WPB composite samples for different mix ratios studied. The results showed an increase in UPV as the content of quarry dust increased. The significant increment of 44.3, 53, and 64.4 % for P&QD 1:1, P&QD 1:2, and P&QD 1:3 respectively, compared to that of P&QD 1:0. The lesser UPV value of P&QD 1:0 is due to the large presence of voids created by the evaporation of air during cooling as seen in the SEM images. However, the quarry dust tends to fill the voids and which explained the higher values of UPV for P&QD 1:1, 1:2, and 1:3. Belmokaddem et al. (2020) reported that the presence of voids decreases the velocity of the pulse due to the near effect of air. Some of the incident wave passing the samples gets partially reflected and the remainder is transmitted, which leads to a reduction of velocity. The relationship between UPV and compressive strength of WPB composites is shown in Figure 11. The finding showed a good correlation between the compressive strength and the UPV of the WPB composites. It can be observed that the compressive strength of the WPB composite increases as the UPV increases. Albano et al. (2009) reported that the elastic properties of medium and volumetric concentrations of components determine the wave velocity. Therefore, compressive strength can be estimated or predicted from the ultrasonic velocity, in a way that destructive tests can be exempted. Furthermore, according to Azhdarpour et al. (2016), PET plastic reduces the speed of sound wave propagation. It is therefore possible that the WPB composite would be suitable as a sound insulator to decrease transmission of acoustic waves.

### 3.6 Influence of material composition on dynamic modulus of elasticity

**Figure 12:**
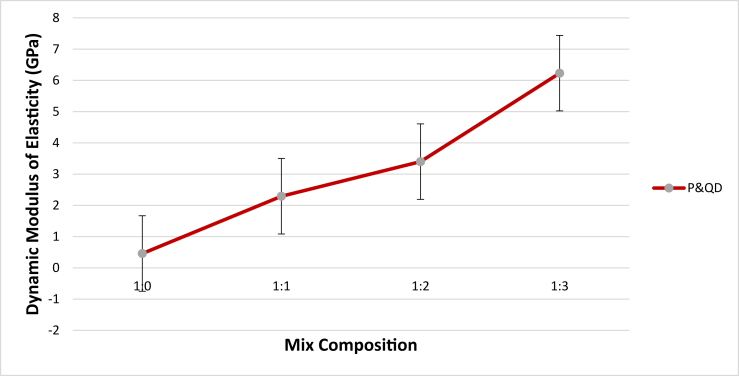
Dynamic modulus of elasticity of WPB composite.

**Figure 13:**
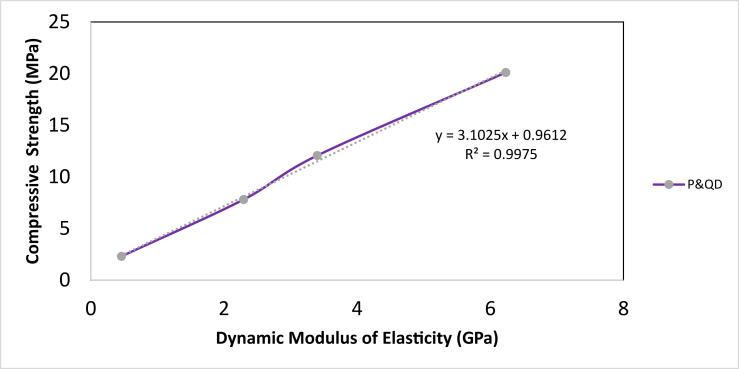
Relationship between dynamic modulus of elasticity and compressive strength of WPB composite.

Figure 12 shows the dynamic modulus of elasticity of the WPB composite against the mix compositions. It can be observed that the variation of the dynamic modulus of elasticity is a function of the content of quarry dust in the composite. This can be attributed to the lower young's modulus of waste PET plastic compare to quarry dust which is a natural mineral. PET plastic has modulus of elasticity between 2.1–3.1 GPa while Quarry dust has 70 GPa (Jacob 2018). The lower value of dynamic modulus of elasticity of P&QD 1:0 sample is expected and the presence of quarry dust in P&QD 1:1, 1:2, & 1:3 sample would significantly increase the dynamic modulus of elasticity. Similar trend was reported by Azhdarpour et al. (2016); Ferreira et al. (2012); Gesoglu et al. (2017); Hannawi et al. (2010); and Yang et al. (2015), the modulus of elasticity of concrete decreases as the addition of plastic aggregate in the concrete mix increases. The modulus of elasticity of the individual materials has an appreciable effect on the modulus of elasticity of the composite (Mohammed et al., 2019); since the deformation of composite material is influenced by the elastic deformation of aggregates. Gesoglu et al. (2017) opined that lower density composite materials would have a lower modulus of elasticity. This may be potentially interesting for certain applications, where materials flexibility and low elastic modulus are perceived to be advantageous such as pavement (Belmokaddem et al., 2020). Figure 13 depicts the relationship between the compressive strength and dynamic modulus of elasticity. As shown, there are linear trends of compressive strength as the dynamic modulus of elasticity increases. The finding shows a good correlation with an R^2^ value of 0.99.

## 4 Conclusions

Ways of mitigating the menace caused by the abundance of waste plastic generated have been a global concern. This study examined the influence of material composition on the morphology and engineering properties of waste plastic binder composite for construction applications. The polyethylene terephthalate type of plastic (PET) was melted and mixed with quarry dust (QD) at different compositions of 1:0, 1:1, 1:2, and 1:3 respectively. From the study, the following conclusions were drawn;

i. The morphological characteristics of WPB composite showed more presence of pores for P&QD 1:0. The pores become fewer for P&QD 1:1, 1; 2, and 1; 3 with the incorporation of quarry dust as it fills the voids.
ii. The WPB composite of P&QD 1:3 gave the highest compressive strength of 20.1 MPa and which meets up with ACI standard minimum requirements for structural lightweight concrete. Therefore, the WPB composite can be considered an alternative material for structural applications, such as pavers in walkways construction.
iii. The WPB composite showed better water absorption property, which is far less than 10%. The hydrophobic waste plastic shielded the pores of the quarry dust from the percolation of water. Hence, the WPB composite will be applicable for water retaining structures.
iv. There is a linear trend of the relationship between UPV and compressive strength of the WPB composites and which gave a good correlation with an R^2^ value of 0.97.
v. It will be crucial to take into account the characteristics of waste plastic composite made from different types of plastic, such as polyethylene (PE), in subsequent research that are connected to the findings of this work.

## Declarations

### Author contribution statement

Yusuf Babatunde: Conceived and designed the experiments; Performed the experiments; Analyzed and interpreted the data; Contributed reagents, materials, analysis tools or data; Wrote the paper.

John Mwero, Raphael Mutuku: Conceived and designed the experiments; Analyzed and interpreted the data; Contributed reagents, materials, analysis tools or data; Wrote the paper.

Yinusa Jimoh: Conceived and designed the experiments; Contributed reagents, materials, analysis tools or data; Wrote the paper.

Daniel Oguntayo: Performed the experiments; Analyzed and interpreted the data; Contributed reagents, materials, analysis tools or data; Wrote the paper.

### Funding statement

This research did not receive any specific grant from funding agencies in the public, commercial, or not-for-profit sectors.

### Data availability statement

Data included in article/supp. material/referenced in article.

### Declaration of interest's statement

The authors declare no conflict of interest.

### Additional information

No additional information is available for this paper.
